# LPA_1_, LPA_2_, LPA_4_, and LPA_6_ receptor expression during mouse brain development

**DOI:** 10.1002/dvdy.23

**Published:** 2019-03-27

**Authors:** Olga Suckau, Isabel Gross, Sandra Schrötter, Fan Yang, Jiankai Luo, Andreas Wree, Jerold Chun, David Baska, Jan Baumgart, Kuniyuki Kano, Junken Aoki, Anja U. Bräuer

**Affiliations:** ^1^ Institute of Cell Biology and Neurobiology, Center for Anatomy Charité–Universitätsmedizin Berlin Berlin Germany; ^2^ Institute of Anatomy, Universitätsmedizin Rostock Rostock Germany; ^3^ Research Group Anatomy School of Medicine and Health Sciences, Carl von Ossietzky University Oldenburg Oldenburg Germany; ^4^ Albrecht Kossel Institute for Neuroregeneration Rostock University Medical Center Rostock Germany; ^5^ Department of Molecular and Cellular Neuroscience, The Scripps Research Institute La Jolla California; ^6^ Translational Animal Research Center University Medical Center of the Johannes Gutenberg–University Mainz Mainz Germany; ^7^ Graduate School of Pharmaceutical Science University of Tokyo Tokyo Japan; ^8^ Research Center for Neurosensory Science Carl von Ossietzky University Oldenburg Oldenburg Germany

**Keywords:** G protein–coupling receptor, growth cone, lysophosphatidic acid, microtubule, primary brain cells, qRT‐PCR

## Abstract

**Background:**

LPA is a small bioactive phospholipid that acts as an extracellular signaling molecule and is involved in cellular processes, including cell proliferation, migration, and differentiation. LPA acts by binding and activating at least six known G protein–coupled receptors: LPA_1–6_. In recent years, LPA has been suggested to play an important role both in normal neuronal development and under pathological conditions in the nervous system.

**Results:**

We show the expression pattern of LPA receptors during mouse brain development by using qRT‐PCR, in situ hybridization, and immunocytochemistry. Only *LPA*_*1*_, *LPA*_*2,*_ *LPA*_*4,*_ and *LPA*_*6*_ mRNA transcripts were detected throughout development stages from embryonic day 16 until postnatal day 30 of hippocampus, neocortex, cerebellum, and bulbus olfactorius in our experiments, while expression of *LPA*_*3*_ and *LPA*_*5*_ genes was below detection level. In addition to our qRT‐PCR results, we also analyzed the cellular protein expression of endogenous LPA receptors, with focus on LPA_1_ and LPA_2_ within postnatal brain slices and primary neuron differentiation with and without cytoskeleton stabilization and destabilization.

**Conclusions:**

The expression of LPA receptors changes depends on the developmental stage in mouse brain and in cultured hippocampal primary neurons. Interestingly, we found that commercially available antibodies for LPA receptors are largely unspecific.

## Key Findings

- LPA1, ‐2, ‐4, and ‐6 genes are dynamically expressed during postnatal brain development.
- LPA1, ‐2, ‐4, and ‐6 genes are differently expressed in the hippocampus, neocortex, cerebellum, and bulbus olfactorius.
- LPA1 and ‐2 gene expression alters during neuronal differentiation.
- LPA1, ‐2, ‐3, ‐4, and ‐6 genes are expressed in glia cells, but differed in gene expression levels.

## 1 INTRODUCTION

Lysophosphatidic acid (LPA) is a bioactive phospholipid present in biological fluids and tissue, including brain.^1^, ^2^, ^3^, ^4^ LPA induces a wide range of cellular responses on primary brain cells and neural progenitors, including intracellular calcium mobilization,^5^, ^6^, ^7^ growth cone collapse and neurite retraction,^1^, ^8^, ^9^, ^10^ cell survival and apoptosis,^11^, ^12^, ^13^ cell proliferation and differentiation,^5^, ^12^, ^14^, ^15^, ^16^ altered postmitotic neuronal migration,^17^ and myelination in the central nervous system (CNS).^16^, ^18^, ^19^, ^20^, ^21^

LPA‐induced effects are mediated through binding to and activation of a specific class of G protein–coupled receptors (GPCRs). To date, six cell‐surface receptors have been identified: LPA_1_ (EDG2), LPA_2_ (EDG4), LPA_3_ (EDG7), LPA_4_ (GPR23/P2Y9), LPA_5_ (GPR92), and LPA_6_ (P2Y5).^3^, ^22^, ^23^, ^24^, ^25^, ^26^, ^27^, ^28^, ^29^ Activated LPA receptors couple with several types of G proteins to trigger a wide range of different downstream signaling pathways: for example, activation of phospholipase C, Rho, Akt, and phosphatidylinositol 3–kinase pathways, or inhibition of adenylyl cyclase.^26^, ^30^, ^31^, ^32^, ^33^ This in turn mediates the cellular responses as described above. The LPA‐induced effects may result from differences in concentration and differential expression of various LPA receptor subtypes. The LPA receptor gene products are detectable in most mammalian tissues in spatial and temporal expression patterns, but a systematic analysis in the developing brain of all six LPA receptors is not yet available.

However, extracellular LPA levels or other phospholipids are known to increase in response to brain injury.^5^, ^34^, ^35^, ^36^ Furthermore, changes in LPA concentration and LPA receptor or LPA metabolic enzyme expression have been linked to neurological disorders such as schizophrenia,^37^, ^38^ cancer growth, metastasis,^32^, ^39^, ^40^ and neuropathic pain.^41^, ^42^ These observations underline the importance of LPA signaling both in normal development and under pathological conditions in the nervous system and suggest that LPA receptors could be drug targets for therapeutic intervention.

Although a large number of publications on LPA receptor gene expression is available, only one study, results of which were published by Ohuchi et al,^43^ has begun systematic analysis of *LPA*_*1–5*_ transcript distribution during mouse organogenesis. Previous publication partly shows discrepancy of *LPA* receptor gene expression, which may be the result of different detection methods. Unfortunately, LPA receptor expression at protein level is unknown due to the lack of specific antibodies.^44^

Our study examined the expression pattern of *LPA*_*1–6*_ receptor transcripts in different mouse brain areas by using different molecular biological techniques to determine gene regulation from late embryonic developmental stages to adulthood. In this phase of life, neurogenesis is almost completed, and astrogenesis and oligodendrogenesis start. During the first postnatal weeks, axons and dendrites continue to grow and mature, followed by synapse formation, maturation, and stabilization.^45^, ^46^ It has been shown that in all these processes, LPA plays an important role, such as in timing of outgrowth, cell migration, myelination, cell survival, and modulating synaptic function.^47^

Furthermore, we aimed to identify specific LPA receptor antibodies using multiple specificity tests. Therefore, for the first time we were able to show the protein expression dynamics of LPA receptors on cellular and subcellular levels.

## 2 RESULTS

### 2.1 *LPA*_*1*_, *LPA*_*2*_, *LPA*_*4*_, and *LPA*_*6*_ receptors predominate and are dynamically expressed during mouse brain development

**Figure 1:**
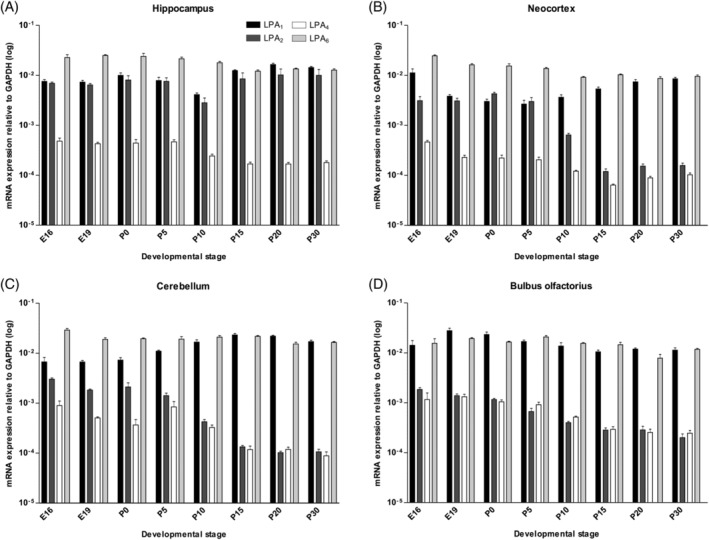
Gene expression profile of *LPA* receptors during mouse brain development. Analysis of *LPA_1‐6_* receptor gene expression in hippocampus (A), neocortex (B), cerebellum (C), and bulbus olfactorius (D) between E16 and P30. The expression levels of each receptor transcript for each sample were normalized to GAPDH. E, embryonic day; P, postnatal day. Error bars represent SD (n = 3)

The group of Dr Noji^43^ reported on the gene expression pattern of *LPA*_*1–5*_ receptors in whole mouse embryos from embryonic day 8.5 (E8.5) to E12.5, which they determined using whole‐mount in situ hybridization (ISH) technique. We used their research as the basis for our study, extending the analysis to the time period from E16 to postnatal day 30 (P30), when astrogenesis, oligodendrogenesis, axon and dendrite outgrowth, and synapse formation take place. We also included the novel LPA receptor LPA_6_ in our analysis_._ Gene expression of the six *LPA* receptors was analyzed in hippocampus, neocortex, cerebellum, and bulbus olfactorius using quantitative real‐time PCR (qRT‐PCR) (Figure 1). Overall, while dynamically expressed, *LPA*_*1*_, *LPA*_*2*_, *LPA*_*4*_ *,* and *LPA*_*6*_ (Figure 1A–D) were detected throughout all developmental stages and in all brain regions tested, as described in more detail below; *LPA*_*3*_ and *LPA*_*5*_ expression remained below detection level (Figure 1A–D).

#### 2.1.1 Hippocampus

The hippocampal region exhibited dynamic expression of *LPA*_*1*_, *LPA*_*2*_, *LPA*_*4*_, and *LPA*_*6*_ receptor transcripts (Figure 1A). Throughout all analyzed developmental stages, *LPA*_*1*_ *, LPA*_*2*_ *,* and *LPA*_6_ expression was at least 10‐fold higher than *LPA*_*4*_ receptor transcripts (Figure 1A). Only in the hippocampus were *LPA*_*1*_ *, LPA*_*2*_ *,* and *LPA*_*6*_ receptors almost constitutively expressed during development.

#### 2.1.2 Neocortex

The *LPA*_*1*_ receptor was present at almost the same level in neocortical tissue as in the hippocampus throughout the investigated developmental stages (Figure 1B). Over time, a slight U‐type course with a minimum gene expression around birth could be detected (Figure 1B). *LPA*_*2*_ transcripts showed no changes in expression at embryonic stages up to P5. After P5, the *LPA*_*2*_ receptor showed a strong down‐regulation (up to 10‐fold) until P15 and then remained stable at this low level until P30 (Figure 1B). The *LPA*_*4*_ receptor decreased slightly from E16, reaching its minimum at P15. At P20 and P30, the expression of *LPA*_*4*_ receptor rose again slightly (Figure 1B). The *LPA*_6_ receptor was almost constitutively expressed, as found in the hippocampus (Figure 1B).

#### 2.1.3 Cerebellum

In cerebellum, the *LPA*_*1*_ transcript level showed weak up‐regulation after birth and peaked at P15 (Figure 1C). In contrast, *LPA*_*2*_ and *LPA*_*4*_ transcripts decreased consistently over time, with the exception of P5, where *LPA*_*4*_ showed an up‐regulation to the level of E16. At E16 and E19, the expression of *LPA*_*2*_ transcripts was 5‐fold weaker compared to that of *LPA*_*4*_. After birth the two receptors showed equal expression pattern, and from P15 the transcripts remained stable (Figure 1C). The *LPA*_6_ receptor was almost constitutively expressed, similar to hippocampus and neocortex (Figure 1C).

#### 2.1.4 Bulbus olfactorius

*LPA*_*1*_ mRNA expression was high in bulbus olfactorius and increased slightly at stages E19 and P0 (Figure 1D). The expression was at least 10‐fold higher (at E16) than that of *LPA*_*2*_ or *LPA*_*4*_ receptors throughout all analyzed developmental stages, with the greatest difference in values at P30. The gene levels of *LPA*_*2*_ and *LPA*_*4*_ receptors were similar and were consistently down‐regulated between E16 and P30 (Figure 1D). Of all brain areas analyzed, bulbus olfactorius exhibited the lowest expression of *LPA*_*2*_ and *LPA*_*4*_ throughout all investigated development stages (Figure 1D). Again, the *LPA*_6_ receptor mRNA was almost constitutively expressed in the same expression level as *LPA*_1_ (Figure 1D).

### 2.2 Cellular localization of *LPA*_*1*_, *LPA*_*2*_, *LPA*_*4*_, and *LPA*_*6*_ receptor mRNA in postnatal stages of different mouse brain areas

**Figure 2:**
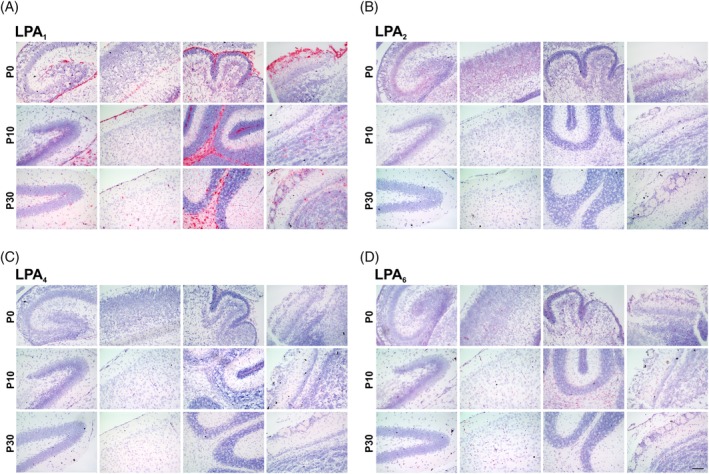
In situ hybridization of *LPA* receptors during mouse brain development. Analysis of *LPA1* (A), *LPA2* (B), *LPA4* (C), and *LPA6* (D) receptor mRNA expression at P0 (first row), P10 (second row), and P30 (third row) in hippocampus formation or dentate gyrus (first column), neocortex (second column), cerebellum (third column), and bulbus olfactorius (fourth column) is shown in red. Nuclei were counterstained with hematoxylin (blue). P, postnatal day. Scale bar = 100 μm

For verification of qRT‐PCR results and cellular localization, we performed ISH of *LPA*_*1*_ *, LPA*_*2*_ *, LPA*_*4*_ *,* and *LPA*_*6*_ receptors in hippocampus/dentate gyrus, neocortex, cerebellum, and bulbus olfactorius in P0, P10, and P30 (Figure 2) developmental stages.

*LPA*_*1*_ mRNA was detected in all examined brain areas and all developmental stages in high amounts, with very strong signals in white matter regions such as the corpus callosum and arbor vitae of the cerebellum, where oligodendrocytes predominantly are located, as well as in compact neuronal structures like the dentate gyrus of the hippocampus (Figure 2A). Also, in non‐neuronal cells, the *LPA*_*1*_ mRNA expression was detectable, like in leptomeninges, covering the neocortex.

A decrease in *LPA*_*2*_ mRNA during development could be observed in all examined regions, as well as in the hippocampus, where qRT‐PCR did not show developmental mRNA decrease (Figure 2B). However, the *LPA*_*2*_ mRNA signal in particular is detectable in compact neuronal structures.

As indicated by qRT‐PCR, *LPA*_*4*_ signal was detected only weakly throughout the brain, with a slight decrease in more mature stages, but still detectable in compact neuronal structures, like the dentate gyrus (Figure 2C).

*LPA*_*6*_ receptor mRNA showed weaker signal then expected from qRT‐PCR results but was clearly detectable throughout all examined areas and stages. ISH confirmed its continuous and overall expression during mouse brain development and showed signals in compact neuronal regions, as well as in brain regions where non‐neuronal cells are mainly located, such as white matter of the arbor vitae of the cerebellum or leptomeninges (Figure 2D).

### 2.3 LPA_1_ and LPA_2_ receptor protein expression and N‐glycosylation in mouse brain

Many commercially available antibodies against synthetic peptides corresponding to regions of human or mouse LPA receptor proteins have never been analyzed for their specificity.^48^, ^49^ We have comprehensively evaluated seven commercial antibodies against LPA_1_, LPA_2_, and LPA_4_ proteins using full‐length mouse or human (LPA_1_ mouse to human, 97% identity; LPA_2_ mouse to human, 84% identity) expression constructs overexpressed in HEK293 cells, wild‐type (WT), and null mouse tissue lysates.

**Figure 3:**
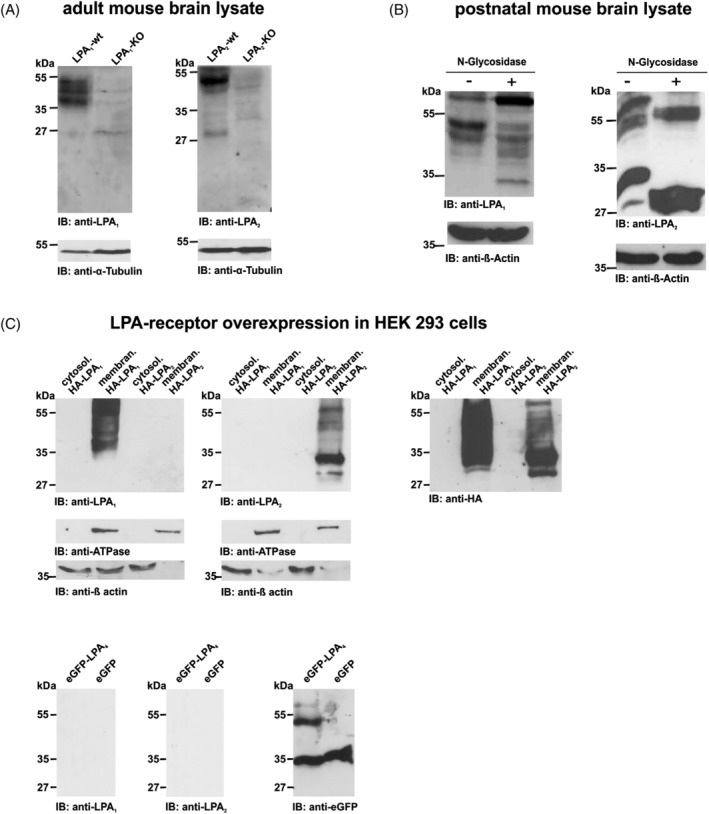
Specificity tests of anti‐LPA1 and anti‐LPA2. A: Whole brain lysates from LPA1‐KO, LPA2‐KO, and WT mice were separated on SDS‐PAGE. One triple band between 55 and 35 kDa in WT mice exhibited anti‐LPA1, whereas no band pattern was detectable in LPA1‐KO lysates. Anti‐LPA2 showed a dominant band pattern around 55 to 45 kDa and a weaker band around 27 kDa in WT, whereas no band pattern was detectable in LPA2‐KO lysates. In conclusion, both LPA receptor antibodies were able to specifically recognize their endogenous mouse protein target. Alpha‐tubulin was used as loading control. B: Postnatal mouse brain lysates were probed with anti‐LPA1 or anti‐LPA2 before and after incubation with N‐glycosidase F. Deglycosylation of LPA1 led to a clear shift, with bands detected around 30 kDa. Endogenous glycosylated protein also showed an LPA1‐positive band around 70 kDa. For LPA2, N‐glycosidase F treatment resulted in a clear shift, with bands detected between 55 and 27 kDa. These results indicate N‐glycosylation of both receptors. Beta‐actin was used as loading control. C: Representative western blots of cytosol and membrane protein lysates from HEK293 cells overexpressing HA‐LPA1, HA‐LPA2, or eGFP‐LPA4 constructs were used to exclude cross‐reactions. Both antibodies also showed specificity to their target proteins in this test. Anti‐HA, anti‐GFP, and anti‐ATPase were used as loading controls. KO, knockout, WT, wild‐type

Most antibodies used for immunoblotting were not able to detect overexpressed mouse or human LPA_1_, LPA_2_, or LPA_4_ proteins and moreover exhibited a strong background signal. No endogenously expressed proteins were recognized by most tested antibodies in different types of mouse tissue. Furthermore, the antibodies recognized cross‐reactivity bands with other proteins in WT and LPA_1_‐ or LPA_2_‐deficient KO protein lysates (data not shown). From seven existing commercially available antibodies, only one was specific for LPA_1,_ as shown in Figure 3A, C. Anti‐LPA_2_, which was generated and kindly donated by J. Aoki, was also specific (Figure 3A, C).

Both antibodies were shown to be sensitive, as they detected the LPA_1_ or LPA_2_ protein 1) when endogenously present in mouse brain protein lysates (Figure 3A, B), and 2) when fused to HA (HA‐LPA_1_ or HA‐LPA_2_) and overexpressed in HEK293 cells (Figure 3C). Specifically, western blot analysis showed bands between 55 and 35 kDa in native adult brain lysates and in HA‐tagged human LPA_1_ overexpressed in HEK293 cells lysate. The bands match the in silico predicted molecular weight of 41 kDa for LPA_1_ (ExPASy; http://www.expasy.ch/tools) and were not detectable in brain lysates from LPA_1_‐KO mice (Figure 3A).

Immunoblot analyses for LPA_2_ showed a dominant band pattern around 55 to 45 kDa and around 27 kDa in native brain lysate and were not detectable in brain lysates from LPA_2_‐KO mice (Figure 3A). The bands match the in silico predicted molecular weight of 39 kDa for LPA_2_ (ExPASy; http://www.expasy.ch/tools).

To assess post‐translational modification, N‐glycosylation of both receptors was enzymatically removed by N‐glycosidase F, and both antibodies were found to be sufficient to detect deglycosylated protein. Deglycosylation of LPA_1_ produced a dominant band at 70 kDa and a new band around 30 kDa compared to glycosylated, endogenous protein (Figure 3B). For LPA_2_, deglycosylation led to a clear shift, with bands detected between 55 and 27 kDa (Figure 3B). The immunoblots showed a different signal for overexpressed full‐length HA‐LPA_1_ and HA‐LPA_2_ compared to endogenous protein from mouse brain lysate. This band pattern could be explained as a consequence of post‐translational modification of endogenous LPA_1_ and LPA_2_ receptors.

Additionally, anti‐LPA_1_ and anti‐LPA_2_ did not detect eGFP‐LPA_4_ fusion protein in immunoblotting, which was further evidence of the antibodies' specificity (Figure 3C).

**Figure 4:**
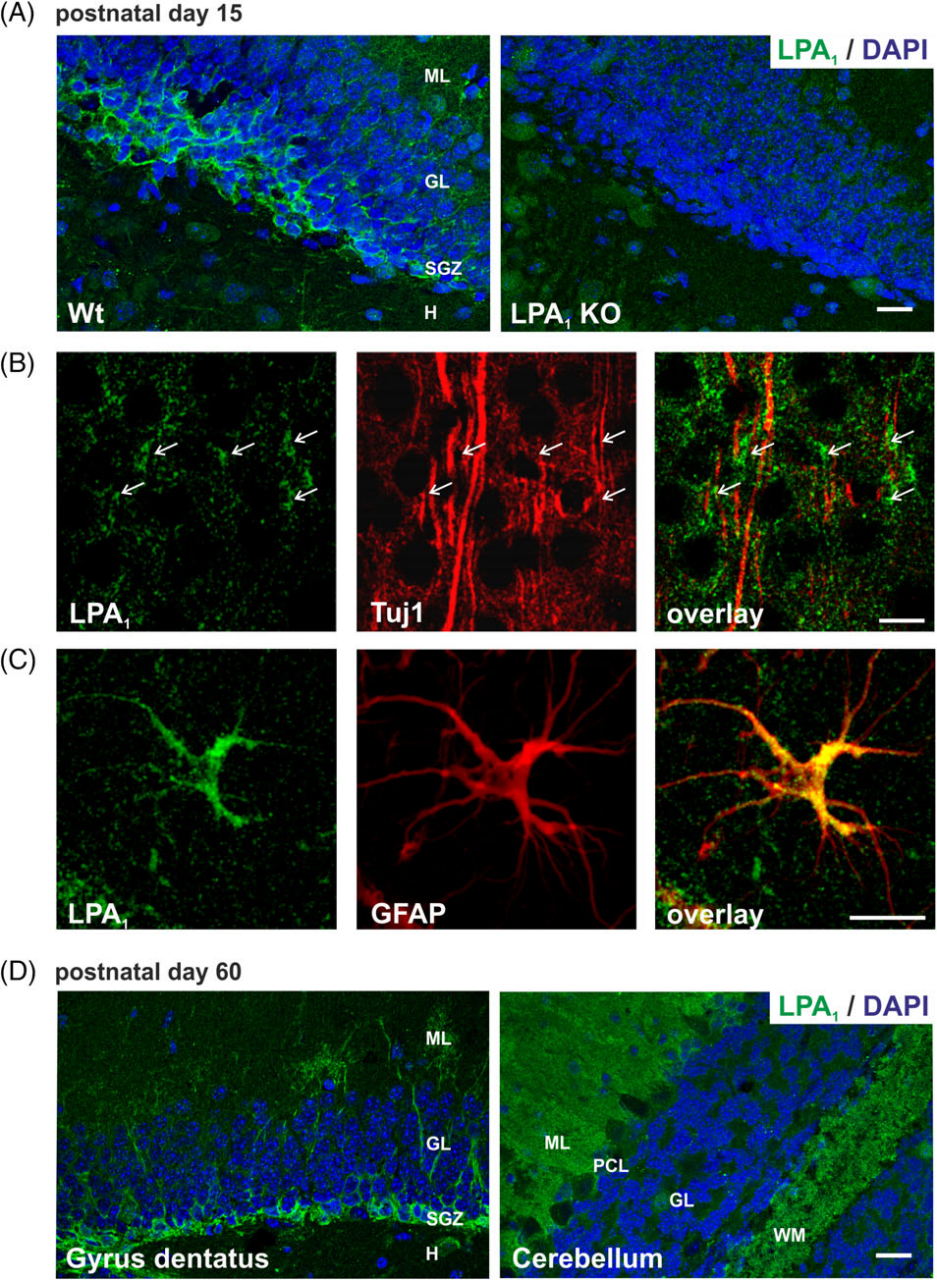
LPA1 receptor protein expression in mouse brain. A: LPA1 antibody specificity is verified by immunostaining of WT and LPA1 KO mice brains at P15. Representative confocal images of LPA1 (green) expression in WT dentate gyrus shows high LPA1 protein expression in neuroblasts of the SGZ, whereas staining is not detectable in KO mice. B: Neurons were visualized with the specific marker for postmitotic immature neurons Tuj1 (red). Cell bodies of neocortical neurons exhibited LPA1 receptor expression (green, arrows). C: Representative confocal image of LPA1 double‐stained with the astrocyte marker GFAP (red). LPA1 receptor is expressed in the cell soma and in astrocyte processes. D: Representative confocal images of LPA1 (green) expression in mouse dentate gyrus and cerebellum at P60 is shown by immunohistochemistry. GL, granular layer; H, hilus; KO, knockout; ML, molecular layer; P, postnatal day; SGZ, subgranular zone; WM, white matter; WT, wild‐type. Scale bars A,D = 20 μm. Scale bar C = 10 μm

**Figure 5:**
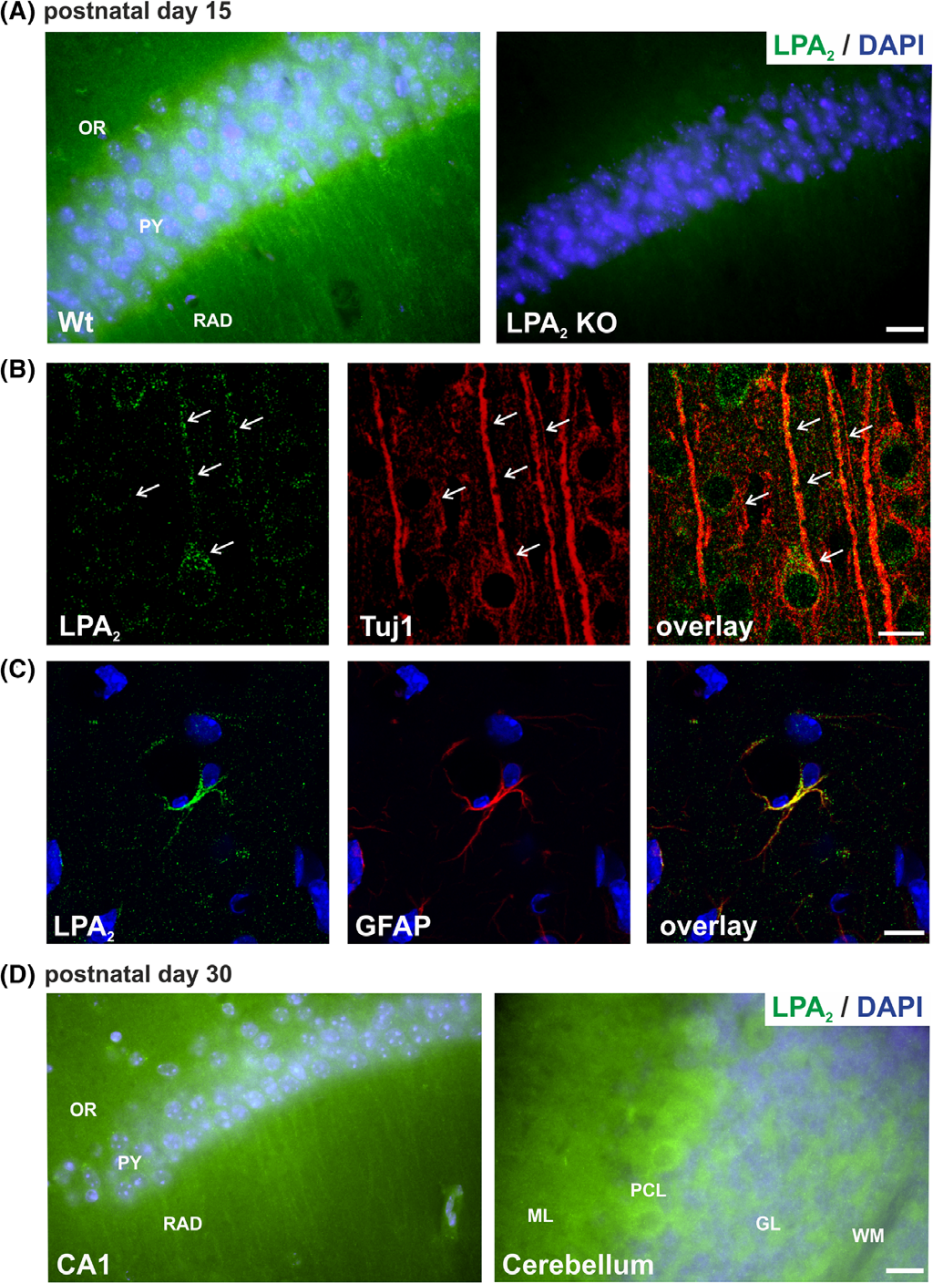
LPA2 receptor protein expression in mouse brain. A: LPA2 antibody specificity is verified by immunostaining of WT and LPA2 KO mice brains at P15. Representative fluorescence images of LPA2 (green) expression in WT CA1 region of the hippocampus shows punctuated LPA2 protein expression in somata and dendrites of pyramidal CA1 neurons, whereas staining is not detectable in KO mice. B: Neurons were visualized with the specific marker for postmitotic immature neurons Tuj1 (red). LPA2 receptor showed a signal in the extensions of Tuj1‐positive neuroblasts (green, arrows). C: Representative confocal images of LPA2 double‐stained with the astrocyte marker GFAP (red). LPA2 receptor is expressed in the cell soma and in astrocyte processes. D: Representative fluorescence images of LPA2 (green) expression in mouse CA1 hippocampal region and cerebellum at P30 is shown by immunohistochemistry. GL, granular layer; H, hilus; KO, knockout; ML, molecular layer; OR, striatum oriens; P, postnatal day; PY, striatum pyramidale; RAD, striatum radiale; SGZ, subgranular zone; WM, white matter; WT, wild‐type. Scale bars A,D = 20 μm. Scale bar C = 10 μm

Because we could not find a specific antibody for the LPA_4_ receptor, we focused subsequent immunohistochemistry experiments on LPA_1_ and LPA_2_ receptors. To analyze the localization of LPA_1_ (Figure 4) and LPA_2_ (Figure 5) in mouse brain, we performed colocalization studies to determine cellular distribution of both receptors.

Western blot results on LPA_1_ and LPA_2_ antibody specificity were confirmed by immunostaining of WT and knockout (KO) brain sections, respectively, of P15 mice (Figures 4A and 5A). LPA_1_ antibody showed strong staining of stem cells of the subgranular zone (SGZ) in the hippocampus, which was not detectable in KO brain sections (Figure 4A). Immunostaining with LPA_2_ antibody was much weaker and showed diffuse background staining, but a punctuated pattern in soma of pyramidal neurons and proximal dendrites in the CA1 region of the hippocampus was clearly detectable and could not be shown in KO brain sections (Figure 5A).

The LPA_1_ protein was barely observable in neuroblasts, expressing the class III β‐tubulin (Tuj1) in P15 mouse cortex (Figure 4B). In contrast, LPA_2_ receptor immunostaining revealed a clear colocalization in Tuj1‐positive neuroblasts (Figure 5B), where the expression was detectable in cell bodies and extensions of cortical neurons (Figure 5B). Detailed immunohistochemistry analyses of LPA_1_ and LPA_2_ receptor expression in astrocytes has shown that both are expressed in the cell soma and in astrocyte processes (Figures 4C and 5C).

Additionally, we examined LPA_1_ and LPA_2_ protein expression in adult WT mice (Figures 4D and 5D). In accordance with qRT‐PCR results, LPA_1_ protein expression, as in P15, was strongly detectable in stem cells of the SGZ of the hippocampus and in white matter of the arbor vitae and molecular layer of the cerebellum of P60 WT mice, confirming the almost constant expression of LPA_1_ during brain development (Figure 4D). LPA_2_ protein was still detectable in P30 mice in soma and proximal dendrites of pyramidal neurons of CA1 hippocampal region and in Purkinje cells of the cerebellum (Figure 5D). However, the diffuse background staining and weak LPA_2_ signal impeded explicit analysis of adult stages but confirmed the decrease of LPA_2_ expression during maturation of the brain, which was shown by qRT‐PCR.

### 2.4 *LPA*_*1*_, *LPA*_*2*_, *LPA*_*4*_, and *LPA*_*6*_ receptors predominate and are dynamically expressed in brain cells

#### 2.4.1 Hippocampal neurons

**Figure 6:**
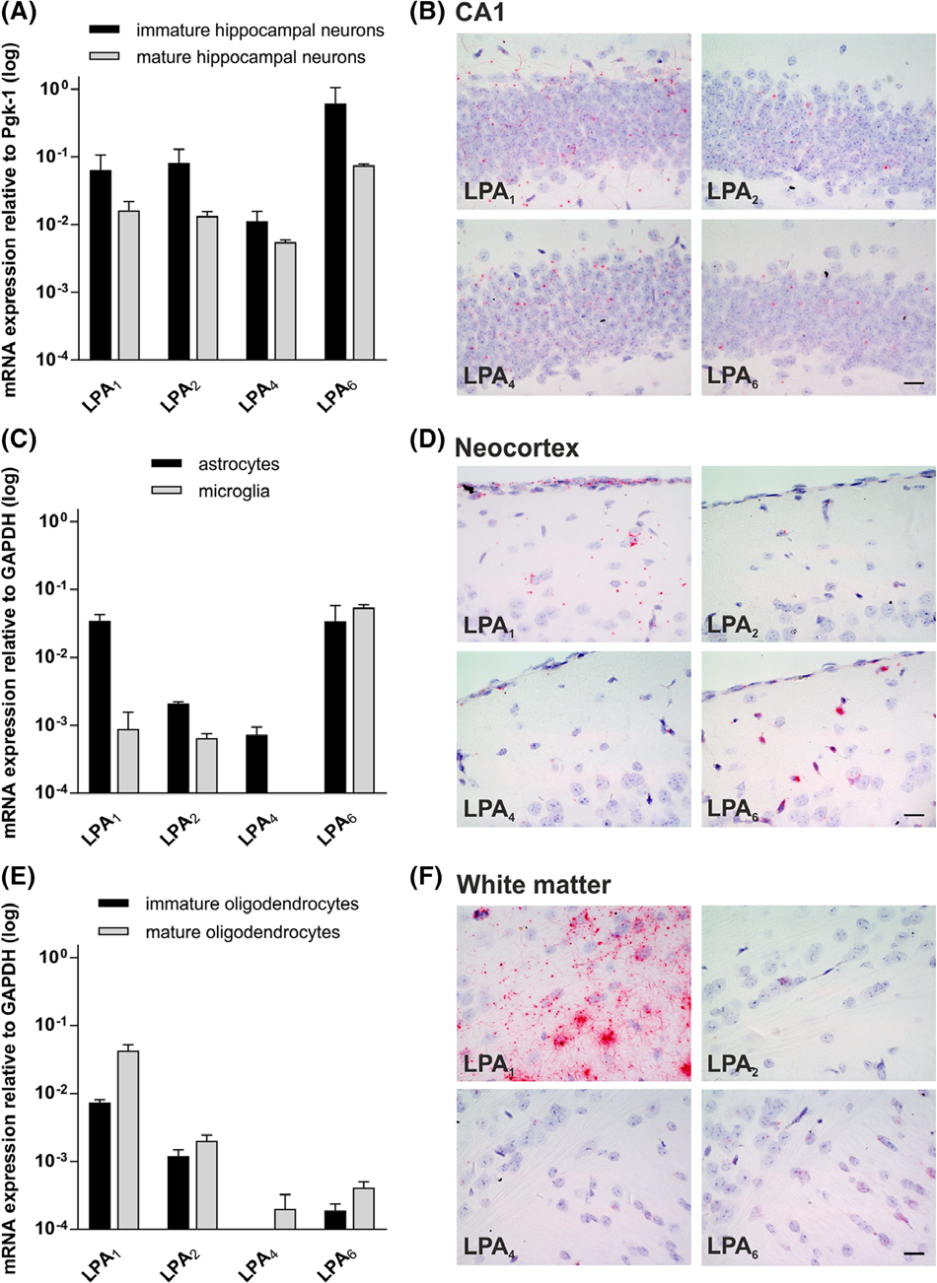
Gene expression profile of *LPA* receptors in brain cells. A: Quantitative RT‐PCR of *LPA* receptor transcripts from immature (2 DIV) and mature (14 DIV) hippocampal neurons. qRT‐PCR analysis reveals a robust expression of *LPA1*, *LPA2*, *LPA4,* and *LPA6* in both immature and mature neurons, whereas *LPA3* and *LPA5* were not detectable. The expression level of each receptor transcript for each sample was normalized to Pgk1. Error bars represent SD (n = 3). B: In situ hybridization of *LPA1*, *LPA2, LPA4*, and *LPA6* receptors in CA1 region of adult (P30) hippocampus. All receptors are expressed in the hippocampal formation in compact neuronal structures like the CA1 region. C: Quantitative RT‐PCR of *LPA* receptor transcripts from primary astrocytes and microglia cells. In cultured primary astrocytes, *LPA1* and *LPA6* expression showed the highest transcript level, while *LPA2* and *LPA4* expression was more than 10 times lower. *LPA3* and *LPA5* were below detection level. In cultured primary microglia cells, only *LPA6* receptor shows a robust expression, whereas *LPA1* and *LPA2* mRNA expression was more than 10 times lower compared to *LPA6. LPA3, LPA4,* and *LPA5* were below detection level. The expression level of each receptor transcript for each sample was normalized to GAPDH. Error bars represent SD (n = 3). D: In situ hybridization of *LPA1*, *LPA2*, *LPA4,* and *LPA6* receptors in the adult (P30) neocortex. Here the receptors are also expressed in non‐neuronal cells, like in leptomeninges covering the neocortex. E: Quantitative RT‐PCR of *LPA* receptor transcripts from immature and mature oligodendrocytes. *LPA1*, *LPA2*, and *LPA6* receptor expression was detectable in immature oligodendrocytes. Here, *LPA1* shows the highest gene expression. In mature oligodendrocytes, *LPA1*, *LPA2*, *LPA4*, and *LPA6* gene expression was detected with a slight increase in expression level compared to immature oligodendrocytes. *LPA3* and *LPA5* expression were below detection level. The expression level of each receptor transcript for each sample was normalized to GAPDH. Error bars represent SD (n = 3). F: In situ hybridization of *LPA1*, *LPA2*, *LPA4*, and *LPA6* receptors in adult (P30) white matter regions of the corpus callosum. *LPA1* receptor mRNA is strongly expressed in white matter areas of the adult mouse brain. *LPA2*, *LPA4,* and *LPA6* receptor mRNA was detected with low signals in cells of the white matter region of the corpus callosum. DIV, days in vitro; P, postnatal day. Scale bars = 20 μm

To further specify the cellular abundance of *LPA*_*1–6*_, we used qRT‐PCR and ISH to analyze its expression in primary cultured brain cells (Figure 6A–F). We used hippocampal neurons after two days in vitro (2 DIV; *immature*). A cell at this maturation stage exhibits long neurites, one of which becomes the axon and elongates very rapidly, while the other neurites do not continue growing. Quantitative RT‐PCR performed on young primary hippocampal neurons showed similar levels of *LPA*_*1*_ and *LPA*_*2*_ transcript expression (Figure 6A). These results corresponded to the *LPA* receptor expression in embryonic hippocampal tissue (Figure 1A). Furthermore, *LPA*_*4*_ receptor expression was more than 10 times lower than that of *LPA*_*1*_ and *LPA*_*2*_, whereas *LPA*_*6*_ was expressed 10 times higher. Additionally, expression of *LPA*_*3*_ and *LPA*_*5*_ in primary neurons was below detection. In hippocampal neurons of 14‐day‐old cultures (14 DIV; *mature*), when an extensive network of synaptic connections has been established, the *LPA*_*1*_, *LPA*_*2*_, and *LPA*_*6*_ expression decreased, whereas the *LPA*_*4*_ receptor decreased only slightly in comparison to immature neurons (Figure 6A). mRNA expression in hippocampal neurons was also confirmed by ISH experiments, where all four tested receptors were detected in the hippocampal formation of adult mice, as shown in the CA1 region in Figure 6B.

#### 2.4.2 Astrocytes and microglia cells

Expression of *LPA*_*1*_ was more than 10‐fold higher in primary astrocytes compared to microglia (Figure 6C). The amount of *LPA*_*1*_ receptor expression in primary astrocytes was similar to the expression level in immature neurons (Figure 6A,B). However, the *LPA*_*2*_ receptor expression was more than 10 times lower in astrocytes and microglia in comparison to neurons, as well as in comparison to *LPA*_*1*_ expression in astrocytes (Figure 6C). *LPA*_*4*_ receptor was detectable in astrocytes only at a low level (Figure 6C). Interestingly, the *LPA*_*6*_ receptor shows similar expression level in astrocytes and microglia with a comparable expression level to mature neurons (Figure 6A–C). All four receptors were detectable by ISH in leptomeninges, indicating their additional non‐neuronal expression in the brain (Figure 6D). Again, expression of *LPA*_*3*_ and *LPA*_*5*_ in primary astrocytes and microglia cells was below detection.

#### 2.4.3 Oligodendrocytes

In primary immature and mature oligodendrocytes, *LPA*_*1*_ receptor showed the highest expression in comparison to the other *LPA* receptors (Figure 6E), with an increase during maturation (Figure 6E). The *LPA*_*2*_ receptor showed a 10‐times‐weaker expression level in comparison to *LPA*_*1*_, with a slight increase during maturation (Figure 6E). *LPA*_*4*_ receptor was only detectable in mature oligodendrocytes at a very weak expression level. The *LPA*_*6*_ receptor expression showed an increase in expression level during maturation, but at a very low expression level compared to *LPA*_*1*_ and *LPA*_*2*_. ISH in white matter area of the corpus callosum of adult mice brain revealed very strong *LPA*_*1*_ receptor mRNA expression and lower expression of *LPA*_*2*_, *LPA*_*4*_, and *LPA*_*6*_ receptors (Figure 6F). Again, expression of *LPA*_*3*_ and *LPA*_*5*_ in primary astrocytes and microglia cells was below detection.

### 2.5 Subcellular distribution of endogenous LPA_1_ and LPA_2_ receptor protein expression alters with neuronal maturation

**Figure 7:**
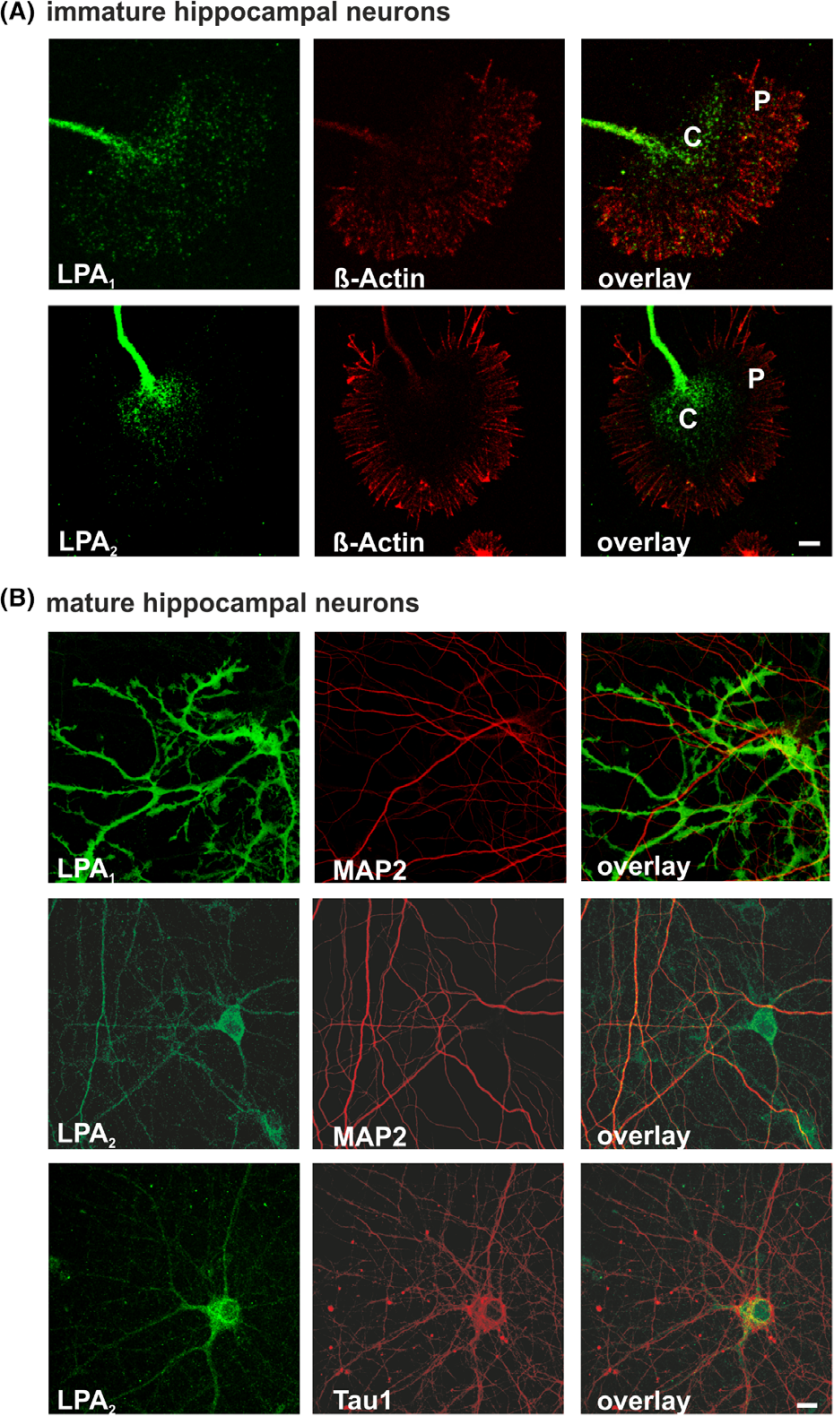
Subcellular distribution of LPA receptors in primary brain cells. Immunocytochemical analysis of endogenous *LPA1* and *LPA2* receptors within immature and mature neurons in vitro. A: Double‐staining was prepared with anti‐ß‐actin (red) to visualize the cytoskeleton of a growth cone from hippocampal neurons cultured 2 DIV. *LPA1* (green) was detected all over the central region of the growth cone, as well as in the lamellipodia and filopodia. *LPA2* (green) was primarily located in the center of the growth cone. B: Representative confocal images of mature primary neurons cultured 14 DIV are shown. Cells were double‐stained with the dendritic marker MAP2 (red) or axonal marker Tau1 (red). *LPA1* receptor (green) was not detectable in mature neurons but was detected strongly in non‐neuronal cells. LPA2 receptor (green) was detected at neuronal cell bodies, weakly in MAP2‐positive dendrites, but clearly in Tau1‐positive axons. C, central region; DIV, days in vitro; P, peripheral region. Scale bars = 10 μm

Young hippocampal neurons showed typical formations at the tips of the axon where anti‐ß‐actin was used to visualize the cytoskeleton of growth cones (Figure 7A). Here, the LPA_1_ receptor was detected as a dotted, weak signal all over the growth cone, from the center region (C‐region) to the actin‐rich peripheral region (P‐region) (Figure 7A). The LPA_2_ receptor was also located in the growth cones, but the signal was predominantly located in the C‐region and not the periphery (Figure 7A). Interestingly, the protein distribution of LPA_1_ and LPA_2_ receptors was equal on the microtubule cytoskeleton, which turns into bundles aligned in parallel to form a nascent neurite shaft tipped by the growth cone (Figure 7A).

LPA_1_ protein was not detectable in mature neurons when an extensive neuronal network had been established but was only observed in non‐neuronal cells (Figure 7B). However, LPA_2_ was clearly detectable in mature neuronal soma and weakly in dendrites, as shown by colocalization with the dendrite marker MAP2. Colocalization with the axonal marker Tau1 protein showed LPA_2_ localization in the proximal axon segment (Figure 7B).

### 2.6 Subcellular distribution of LPA_1_ expression alters upon microtubule stabilization and destabilization

**Figure 8:**
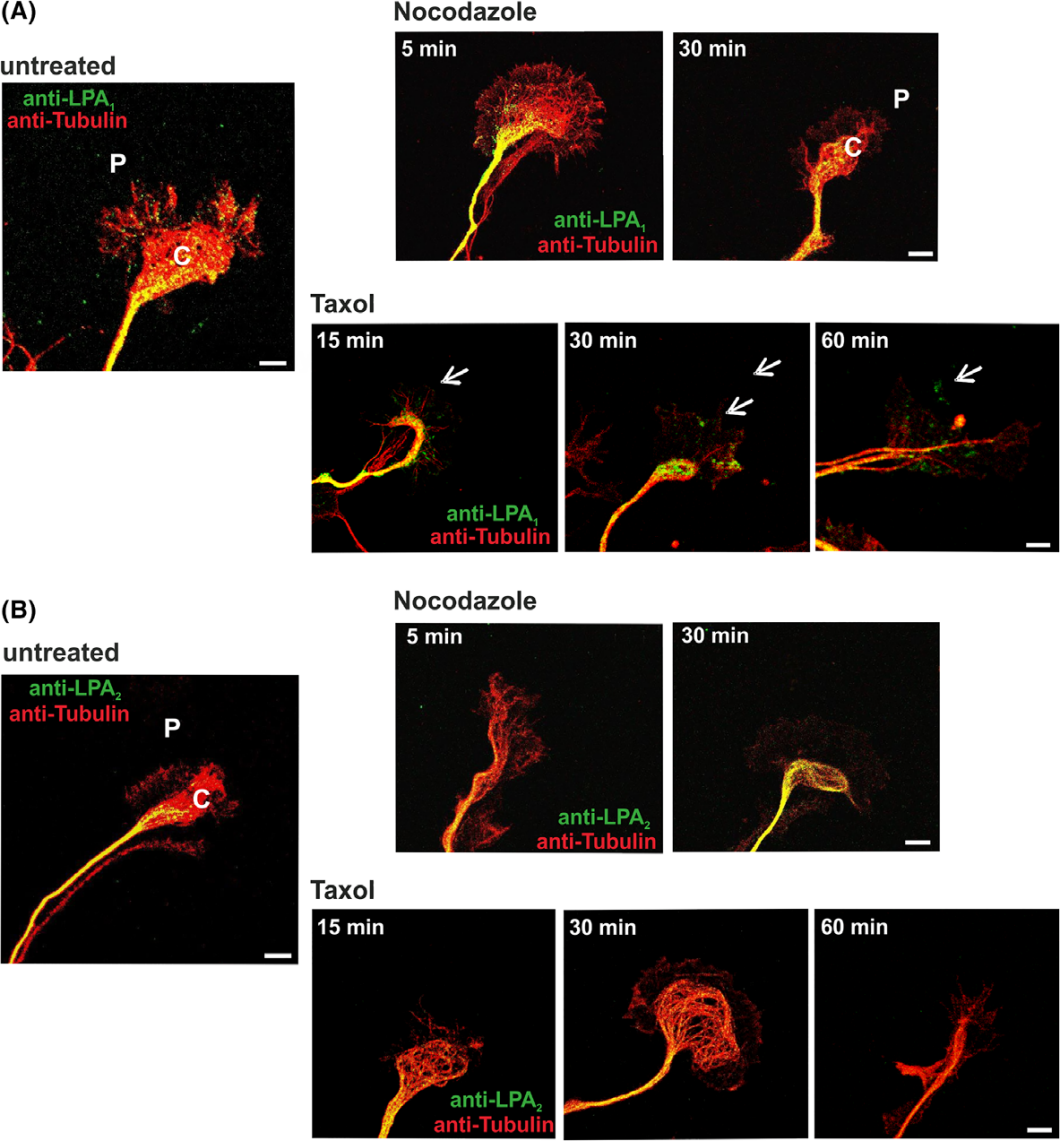
Localization analysis of LPA receptors upon stabilization/destabilization of microtubules. Immunocytochemical analysis of endogenous *LPA1* and *LPA2* receptors within immature neurons in vitro. Double‐staining was prepared with anti‐α‐tubulin (red) to visualize the cytoskeleton of a growth cone from hippocampal neurons 2 DIV. A: *LPA1* (green) was detected in the central and peripheral regions prior to treatment. Treatment with nocodazole led to a restriction of LPA1 expression to the central region and a complete disappearance in the peripheral region. After 15 minutes, Taxol incubation first clusters away from the central region were seen. At the end of the treatment, clusters of *LPA1* were found in the peripheral region of the growth cone. Arrows indicate LPA1 clusters. B: In contrast, *LPA2* (green) was found only in the central region in untreated growth cones. No effect on the *LPA2* expression was observed after nocodazole or Taxol treatment. C, central region; DIV, days in vitro; P, peripheral region. Scale bars = 10 μm

To mimic navigation processes of growth cones during neuronal polarization, we destabilized the microtubule network with nocodazole. Cells were incubated with nocodazole for five minutes and after removal further incubated in medium for 25 minutes. Right after the treatment, a lower LPA_1_ expression in the P‐region in comparison to untreated neurons was found (Figure 8A). After further 25 minutes without nocodazole (30 minutes), LPA_1_ expression concentrated in the C‐region. Hence, it shows that the treatment led to a decrease in growth cone size and a reduction of LPA_1_ protein in the P‐region. To mimic neuronal polarization, the microtubules of neurons were stabilized using Taxol. Taxol treatment led to a slightly changed distribution of LPA_1_ receptors in the growth cone. After 15 minutes and 30 minutes, clusters of LPA_1_ were found in the P‐region of the growth cone (Figure 8A). Taxol was removed after 30 minutes and neurons were incubated in neuronal medium for an additional 30 minutes. At this time point (60 minutes), the LPA_1_ signal was found in clusters in the P‐region similar to the 30‐minute time point (Figure 8A).

In comparison, destabilization of the growth cone by nocodazole did not affect the localization of LPA_2_ (Figure 8B). The treatment did not induce any change in distribution of the receptor or the expression level. Even after the stabilization of the microtubules with Taxol, LPA_2_ expression was found only in the C‐region (Figure 8B). The distribution after this treatment resembles the LPA_2_ expression in untreated neurons, where the receptor is also found only in the C‐region of the growth cone (Figure 7A).

## 3 DISCUSSION

We describe here the expression pattern of the six *LPA*_*1–6*_ receptor genes in mouse brain from late embryonic developmental stage until adulthood. Our results show that during brain development, particularly when astrogenesis and oligodendrogenesis start and axons and dendrites continue to grow and mature, followed by synapse formation, maturation and stabilization, the LPA receptor genes are dynamically regulated in a temporal‐ and spatial‐dependent manner.

### 3.1 LPA receptor gene expression during brain development

Our data of *LPA*_*1*_ receptor expression is in agreement with the results of others, which detected *LPA*_*1*_ transcripts in whole‐brain samples or neocortex of rodents from embryonic until adult stages in a biphasic expression pattern or increased during mouse brain development.^18^, ^34^ Our results revealed that *LPA*_*1*_ mRNA is up‐regulated, down‐regulated, or biphasic‐regulated during brain maturation depending on the analyzed mouse brain area.

The level of *LPA*_*1*_ mRNA in the bulbus olfactorius was high in embryonic stages and down‐regulated after birth. Genetic deletion of *LPA*_*1*_ receptor has been reported as associated with suckling defects,^14^ which could explain the high expression in early stages that we observed.

Studies reporting *LPA*_*1*_ expression in different brain cells vary in their findings during embryonic and postnatal development. Reports have shown *LPA*_*1*_ expression in oligodendrocytes and astrocytes, as well as in neural progenitor cells (NPCs) in the ventricular zone but not in neurons.^16^, ^19^, ^22^, ^50^ Our staining in brain slices of postnatal mice showed a colocalization of LPA_1_ with the astrocyte‐specific marker protein glial fibrillary acidic protein (GFAP), and our qRT‐PCR results demonstrated a high level of *LPA*_*1*_ in cultured astrocytes and oligodendrocytes, whereas cultured microglia cells showed a moderate expression level. Interestingly, it has been shown that LPA‐activated astrocytes can induce neuronal differentiation and axonal outgrowth.^15^, ^51^ Moreover, nerve growth factor (NGF) production can also be enhanced by astrocytes' exposure to LPA.^52^

In oligodendrocytes it has been shown that extracellular LPA levels promote oligodendrocyte differentiation via an increase in Histone Deacetylase 1 and 2 (HDAC1/2) activity.^16^ Two studies show predominant expression of *LPA*_*1*_ and no expression of *LPA*_*3*_ in microglia prepared from mouse brain.^53^, ^54^ In addition to *LPA*_*1*_ *,* we observed the expression of *LPA*_*2*_ in cultured mouse microglia, unlike the reverse transcriptase experiments of Möller et al.^53^ We also found predominant expression of *LPA*_*6*_, which had not previously been tested.

Earlier studies of *LPA* receptor expression showed that *LPA*_*1*_ had the highest expression level of all known LPA receptor genes, as determined by northern blot analyses of human and mouse brain.^22^, ^55^ However, in our study, expression of the *LPA*_*2*_ receptor was equal to that of *LPA*_*1*_ and did not change significantly during hippocampal development. This lends weight to the importance of the *LPA*_*2*_ receptor during all stages in mouse brain development. Furthermore, this data is in line with published work from McGiffert et al, which shows diffuse expression of *LPA*_*2*_ receptor throughout the CNS.^56^ Moreover, we could show a predominant expression of the *LPA*_*2*_ receptor in mature hippocampal neurons. This expression pattern differs from the *LPA*_*1*_ receptor and indicates the possible importance of the *LPA*_*2*_ receptor in neuronal networks. It is known that LPA promotes survival and differentiation of cortical precursor cells^12^ and is involved in the regulation of adult hippocampal precursor cell proliferation and neurogenesis via activation of the AKT and MAPK pathways.^57^

Our qRT‐PCR measurements of *LPA*_*2*_ expression in microglia also differed from previously published data. Whereas Möller et al^52^ could not detect LPA_2_ expression by reverse‐transcriptase PCR in primary mouse microglia, we did observe *LPA*_*2*_ in these cells.

Several studies have analyzed *LPA*_*3*_ expression in tissue with various results. For example, as determined by northern blotting, Bandoh et al could not observe any *LPA*_*3*_ signal in human brain,^24^ whereas another study detected *LPA*_*3*_ mRNA in several areas of the human forebrain, particularly the frontal cortex.^58^ In contrast to the results from Contos et al, which showed *LPA*_*3*_ expression in the mouse brain by northern blot^59^ and RT‐PCR,^60^ we could not detect mRNA transcripts of this receptor by qRT‐PCR at any stage of brain development in the four analyzed brain areas. Previous investigation of assay efficiency and the correlation coefficient for the *LPA*_*3*_ receptor was about 0.95 to 0.99, providing that a data‐interpretation error can be excluded.

Currently available data on *LPA*_*4*_ receptor expression shows a ubiquitous expression pattern in mouse tissue and particularly high levels in ovaries.^43^, ^61^, ^62^ In all tested brain areas and development stages, *LPA*_*4*_ expression levels were much lower than those of *LPA*_*1,*_ *LPA*_*2,*_ and *LPA*_*6*_. To date, no data is available on *LPA*_*4*_ expression in primary cells. By analyzing different primary cell types isolated from mouse brain, we found *LPA*_*4*_ in immature and mature neurons and astrocytes and at a low level in mature oligodendrocytes. LPA_4_ protein is structurally distinct from classical LPA receptors and also binds G proteins. Currently, the physiological role of *LPA*_*4*_ receptor is poorly understood, and *LPA*_*4*_‐deficient mice display no apparent abnormalities.^61^ Because specific antibodies for LPA_4_ are not yet available, intracellular localization and localization studies in the brain have not been possible. Further analyses are required to understand the role of the LPA_4_ receptor in the brain.

Our analysis proves the lack of *LPA*_*5*_ receptor expression in the brain. These results are in line with northern blot data from Lee et al.^26^

LPA_6_ is the most recently identified member of the LPA receptor family.^63^ It has been described that in *Xenopus,* the LPA_6_ receptor is expressed in the developing telencephalon.^64^ These results are in line with ours. We show that the *LPA*_*6*_ transcript is expressed higher than any other LPA receptor in all analyzed mouse brain area and development stages. Moreover, in primary neurons, immature and mature, astrocytes, and microglia we found a prominent *LPA*_*6*_ mRNA expression. Only oligodendrocytes, immature and mature, show only weak *LPA*_*6*_ expression. Especially in microglia, *LPA*_*6*_ receptor shows the highest expression of all LPA receptors. LPA exposure in primary murine microglia leads to a shift toward a proinflammatory M1‐like phenotype.^65^ The regulation of microglia polarization (eg, interference of the LPA signal transmission via LPA receptors) could represent a potential pharmacological target of neuroinflammation in the CNS.

Taken together, our expression analysis of *LPA* receptors throughout developmental and adult mouse brain demonstrated predominant expression of *LPA*_*1*_, *LPA*_*2*_, and *LPA*_*6*_ receptors, indicating a key player role during neuronal development and function. The lack of commercial antibodies specific for LPA receptors makes it difficult to study the expression pattern on protein level. Only one of three tested anti‐LPA_1_ antibodies was specific. The band pattern in the immunoblots correlated with published data by Cervera et al,^20^ who generated a polyclonal antibody against a peptide on the C‐terminus of LPA_1_. No specificity was detected for two anti‐LPA_2_ and two anti‐LPA_4_ antibodies, which are commercially available.

The existence of several bands in the immunoblots points to a putative glycosylation of LPA_1_ and LPA_2_ receptors. Mouse LPA_1_ has 364 AMino acids with an estimated molecular weight of ~41 kDa, whereas mouse LPA_2_ consists of 348 AMino acids with an estimated molecular weight of ~39 kDa (ExPASy; http://www.expasy.ch/tools). Indeed, in silico analysis of the rat, mouse, and human LPA_1_ or LPA_2_ amino acid sequence (NetNGlyc 1.0; http://www.cbs.dtu.dk/services/) revealed a putative consensus N‐glycosylation site of aa 27 and aa 35 for LPA_1_, as well as aa 10 for LPA_2_. N‐glycosylation is the most common glycosylation in proteins, and analysis was done because it is an important post‐translational modification with influence on the functional diversity as well as the activity of a protein. Furthermore, it provides stability for the protein and can affect oligomerization.^66^, ^67^ For Edg‐1, a phospholipid receptor for sphingosine‐1‐phosphate, it has been shown that N‐terminal glycosylation is important for ligand‐induced internalization and receptor dynamics within the membrane, proposing a similar influence for LPA receptors.^68^

### 3.2 Subcellular localization of LPA_1_ and LPA_2_ receptors

For more detailed characterization of LPA receptors, we analyzed the localization of the proteins in primary cultured hippocampal neurons and brain slices. Based on our knowledge from the qRT‐PCR data in primary neurons and the lack of an LPA_4_ and LPA_6_ antibody, we focused this study on LPA_1_ and LPA_2_ receptors alone.

In immature primary neurons, the localization of LPA_1_ and LPA_2_ receptors was especially interesting within the growth cones because the receptor ligand, LPA, has been described as inducing rapid growth cone collapse and neurite retraction in young postmitotic neurons and several cell lines, such as NG108‐15, N1E‐115, PC12, and SY‐SH5Y.^17^, ^22^, ^69^, ^70^, ^71^, ^72^, ^73^, ^74^, ^75^, ^76^, ^77^, ^78^ Primary neurons form a number of immature neurites after plating in neuronal medium, which undergo random growth and retraction processes.^79^ It is possible that LPA receptors are among other proteins involved in the regulation of such minor processes.

LPA_1_ was not only detected in the C‐region of growth cones, but also colocalized with actin‐rich lamellipodia and filopodia. Previous studies of the LPA_1_ homolog in *Xenopus* have shown, for example, that this protein plays a key role in the regulation of normal cortical actin assembly.^80^ The involvement of LPA_1_ in the rearrangement of actin, which is associated with ongoing neuronal polarization, has been shown multiple times.^17^, ^81^ The actin rearrangement induced through G_12/13_ signaling is counteracted by G_q_ signals.^82^ To control those processes, the presence of LPA receptors in the plasma membrane is important. This provides one explanation of LPA_1_ clustering in the actin‐rich P‐region after Taxol treatment, corresponding to the mimicry of advanced polarization because, for example, constitutively active G_q_ can cause massive cell death in neurons.^82^ However, further studies are required to confirm this hypothesis.

In contrast, LPA_2_ was detected in the C‐region but not in the P‐region of growth cones. The C‐region is thicker than the P‐region and is thought to function as a terminal for vesicles and macromolecules transported along axonal microtubules.^83^ Interruption of microtubule dynamics can lead to an abolition of vesicle migration, thus explaining the unchanged LPA_2_ distribution during the stimulation experiments.^84^ LPA_2_ was described as a promoter of cell migration through interaction with the adhesion molecule TRIP6.^85^, ^86^ Localization of LPA_2_ in the C‐region of growth cones, an area where adhesion molecules are located, indicates the role of this receptor during neurite guiding. Furthermore, presynaptically located LPA_2_ has been described as a player in regulation of the glutamatergic transmission.^87^ LPA itself was shown to be an important phospholipid during early cortical development. It stimulates cell proliferation of cortical neuroblasts located in the ventricular zone of the cerebral cortex and inhibits neuronal differentiation in the cortical plate.^88^ Because postmitotic neurons produce LPA, a reciprocal control mechanism may regulate cell proliferation, dendritic outgrowth, and differentiation in these two cortical layers. Our data on primary hippocampal neurons in vitro show localization of LPA_1_ and LPA_2_ receptors in growth cones and may hint at a role in axonal and/or dendritic outgrowth and guidance in the hippocampus. Confirming this approach could be based on LPA_1_ and LPA_2_ receptor expression data in vivo of developmental hippocampus.

The localization pattern of these receptors differs in mature hippocampal neurons in culture compared to immature neurons in that the LPA_1_ receptor could no longer be detected in differentiated neurons. Additionally, LPA_1_ was hardly detected in neurons in brain slices, but instead in GFAP‐positive astrocytes. The localization of LPA_2_ was homogeneously distributed in cell body of mature neurons and Tau1‐positive axons. This expression pattern was in line with LPA_2_ staining in brain slices of postnatal mice.

## 4 CONCLUSION

In this study, we found development‐dependent expression of LPA receptors in mouse brain and in cultured hippocampal primary neurons. In view of the complex expression patterns of LPA receptors within organisms, further physiological and pathophysiological function of LPA signaling remains to be characterized. Currently, six receptors have been identified as specific G protein–coupled receptors for LPA. Recent publications have suggested that three additional receptors, GPR87, GPR35, and P2Y10, may be responsive to LPA.^89^, ^90^, ^91^, ^92^, ^93^ Although further study is required to confirm these observations, it is clear that LPA signaling and LPA receptor expression are very complex. Thus, many more physiological and pathophysiological functions of LPA signaling remain to be revealed and characterized.

## 5 EXPERIMENTAL PROCEDURES

### 5.1 Animals

For all experiments, timed‐pregnant, postnatal, and adult WT C57BL/6 or BALB/c and LPA_1_‐KO or LPA_2_‐KO mice were obtained from Charité–Universitätsmedizin central animal facility (FEM), the central animal facility of the University Medical Center Rostock, or the central animal facility of the Carl von Ossietzky University Oldenburg and were kept under standard laboratory conditions (12‐hour light/dark cycle; 55% +/−15% humidity; 22°C +/− 2°C RT, and water ad libitum, enriched and grouped) in accordance with German and European guidelines (2010/63/EU) for the use of laboratory animals. Approval of experiments was obtained from the local ethics body of Berlin (LAGeSO: T0108/11). For experiments, the day of the vaginal plug following mating was designated E0.5. Experiments were performed on E16, E18, and E19 embryos and perinatal pubs (P0, P5, P10, P15, P20, and P30); 4‐ and 8‐week‐old male mice were used for protein extraction. Generation and characterization of mice with LPA_1_‐ and LPA_2_‐receptor deficiency have been described previously.^14^, ^94^

### 5.2 Primary mouse hippocampal neuron cultures

Primary hippocampal neurons were prepared from E18 (+/−0.5 day) mouse embryos as described previously.^95^ The cells were cultured on poly‐L‐lysine–coated glass coverslips in Neurobasal A medium supplemented with 2% B27, 0.5 mM glutamine (Gibco‐Invitrogen, Basel, Switzerland), and the antibiotics penicillin and streptomycin (100 U/mL PAN‐Biotech, Aidenbach, Germany). Neurons were plated at a density of 2.1 × 10^4^ cells/cm^2^ and were routinely maintained at 37°C and 5% CO_2_.

### 5.3 Primary mouse astrocyte and microglia cultures

For astrocyte and microglia preparation, neocortex and cerebellum from mouse pups aged P0 to P2 were isolated.^34^ The tissue was washed in Dulbecco's Modified Eagle's Medium (DMEM, Gibco‐Invitrogen, Basel, Switzerland) 4.5 g/L glucose containing 10% fetal calf serum (FCS, PAN‐Biotech, Aidenbach, Germany), 200 mM glutamine, and 100 U/mL penicillin/streptomycin, then underwent careful homogenization with a fire‐polished Pasteur pipette and centrifugation at 300 × g and 4°C for one minute. The supernatant was transferred into a new tube, homogenized again, and centrifuged for five minutes at 1200 × g and 4°C. The pellet was re‐suspended in fresh medium and plated in culture flasks, which had been precoated with poly‐L‐lysine. The cells were incubated at 37°C with 5 %CO_2_. After two days, the medium was refreshed and the cells were allowed to continue incubating until most of the microglia had detached from the astrocytes. To remove the remaining attached microglia, the cells were shaken for two hours at 37°C. Afterward, the supernatant was plated again in culture medium containing 2% FCS. The astrocytes still attached to the bottom of the culture flask were used for further experiments after 14 to 16 days.

### 5.4 Primary mouse oligodendrocyte cultures

Oligodendrocytes were prepared after an adapted protocol from Chen et al.^96^ Briefly, the cerebral hemisphere of each mouse at P5 was dissected and then digested with papain (100 U, Worthington Biochemical Corporation) by gentle trituration. The cell suspension from three mice was seeded to a poly‐D‐lysine (100 μg/mL in phosphate‐buffered saline (PBS), Sigma, St. Louis, MO, USA) ‐coated T75 flask and maintained in DMEM high‐glucose medium (Invitrogen, Carlsbad, CA, USA) supplemented with 20% fetal bovine serum (Thermo Fisher, Waltham, MA, USA) and 1× penicillin/streptomycin. After 24 hours, the medium was changed completely, then half of the medium was replaced every three days. Insulin (5 μg/mL, Sigma, St. Louis, MO, USA) was added at DIV 7. At DIV 10, oligodendrocyte progenitor cells (OPCs) were detached from the mixed glial culture by differential shaking (sequentially with 75 rpm for 1 hour and 220 rpm for 16 hours) and purified by adhesion to uncoated Petri dishes. About 200 000 purified cells were seeded into each well of a poly‐D‐lysine‐coated six‐well plate and cultured in Maltose‐Yeast Extract‐Malt Extract (MYM) medium, which contains DMEM high‐glucose medium, sodium pyruvate, L‐glutamine, B27 supplement (all from Invitrogen, Carlsbad, CA, USA), insulin, transferrin, putrescine, progesterone, sodium selenite, T3, hydrocortisone, biotin, vitamin B12 (all from Sigma, St. Louis, MO, USA), and ceruloplasmin (Calbiochem, Merck, Darmstadt, Germany).^97^ OPCs simultaneously differentiate into oligodendrocytes in the absence of mitogens, such as platelet‐derived growth factor (PDGF) and basic fibroblast growth factor (bFGF).^98^ The cells were cultivated without changing the medium and immature oligodendrocytes were collected after three days, mature ones after six days.

### 5.5 Cell culture, transfection, and protein extraction

HEK293 cells were routinely maintained at 37°C and 5% CO_2_ in DMEM supplemented with 10% FCS, 200 mM glutamine, and 100 U/mL penicillin/streptomycin. HEK293 cells were transfected transiently with N‐terminal, HA‐tagged human LPA_1_ and LPA_2_ (in pcDNA3, Invitrogen, Carlsbad, CA, USA) or N‐terminal, eGFP‐tagged mouse LPA_4_ receptor (in pEGFP‐C1, BD Biosciences, Palo Alto, CA, USA). For transfection, cells were seeded at a density of 3 × 10^4^ cells/cm^2^ on poly‐L‐lysine–coated cell culture plates overnight. On the following day, the culture medium was refreshed and 0.52 μg plasmid‐DNA was diluted in 45 μL water, 4 μL CaCl_2_ (2.5 M), and 45 μL HEPES buffer (pH 7.1–7.2). Volume and DNA concentration were calculated to an area of 10 cm^2^ from the cell culture plate. The transfection complexes were added to the cells, and cells were cultured at 37°C and 5% CO_2_ for at least 24 hours. For protein extraction, cells were lysed in lysis buffer (20 mM Tris, pH 7.5, 0.25 M sucrose, 1 mM EGTA, 5 mM EDTA, 25× proteinase inhibitor cocktail from Sigma, St. Louis, MO, USA) and centrifuged for 10 minutes at 13.000 g. The supernatant was replaced and stored at −20°C. For membrane protein isolation, the cells were centrifuged at 150.000 × g for 30 minutes at 4°C. The resulting supernatant held the cytosolic protein fraction and was stored at −20°C. The pellet was re‐suspended in 100 μL lysis buffer and 0.1% Triton X‐100. After further incubation for 30 minutes on ice, it was centrifuged again for five minutes at 5000 × g at 4°C. The supernatant, now containing the membrane proteins, was replaced and stored at −20°C.

### 5.6 Deglycosylation

100 μg protein, 1 μL 25% SDS, and 1 μL ß‐mercaptoethanol (1:50) were boiled for 15 minutes at 65°C. Afterward, 5 μL N‐glycosidase F (5 U) or 5 μL 20 mM Tris (control) were added and incubated at 37°C overnight. 1 μL additional ß‐mercaptoethanol and protein sample buffer were added and an SDS‐PAGE with subsequent western blot and immunodetection were performed.

### 5.7 Immunocytochemistry

Cultured primary neurons were fixed in ice cold 4% paraformaldehyde (PFA) in 1x PBS containing 15% sucrose for 20 minutes at room temperature (RT) and subsequently permeabilized with 0.1% Triton X‐100 and 0.1% sodium citrate in 1x PBS for three minutes at 4°C. Cells were then incubated for two hours at RT in 10% FCS in 1x PBS (PBSF). For stabilization or destabilization studies, the neurons were treated with 100 nM Taxol or 500 nM nocodazole (both from Sigma, St. Louis, MO, USA), respectively, in Hank's Buffered Salt Solution (HBSS, Thermo Fisher, Waltham, MA, USA) for up to 60 minutes. Drug concentration and duration of treatment were adjusted after Witte et al.^99^ After removal of the medium at the indicated time points (5, 15, 30 or 60 minutes) and washing with 1× PBS, cells were fixed in ice cold 4% PFA in 1× PBS containing 15% sucrose for five minutes at RT. At this point cells were kept in 1x PBS to allow synchronization of all wells. Following washing with 1× PBS, they were permeabilized with 0.2% Triton X‐100 in 1× PBS for 10 minutes at RT. Subsequently, cells were incubated with 20% FCS in 1x PBS for two hours at RT. All neurons were stained with polyclonal anti‐LPA_1_ 1:500 (Cayman Chemical, Ann Arbor, MI, USA) or monoclonal anti‐LPA_2_ at 1:250 (generated by J. Aoki), each combined with either monoclonal anti‐ß‐actin 1:500 or monoclonal anti‐MAP2, both diluted to 1:1000 (Sigma, St. Louis, MO, USA), monoclonal anti‐Tau1 at 1:200 (Millipore, Billerica, MA, USA) or monoclonal anti‐α‐Tubulin (1:500, Sigma, St. Louis, MO, USA). All primary antibodies were diluted in PBSF and incubated overnight at RT. Goat anti‐rabbit 488 or goat anti‐rat 488 and goat anti‐mouse 568 Alexa Fluor conjugated (1:1000; Molecular Probes, Eugene, OR, USA) secondary antibodies were diluted in PBSF and incubated at RT for one hour. Coverslips were mounted on slides with Immu‐Mount and used for microscopy.

### 5.8 Immunohistochemistry

C57BL/6, LPA_1_‐KO, and LPA_2_‐KO mice at P15, P30, and P60 were deeply anesthetized with a mixture containing ketamine (Pfizer, Karlsruhe, Germany) and xylazine (Rompun, Bayer HealthCare, Leverkusen, Germany), or with sodium pentobarbital (Narcoren, Boehringer Ingelheim, Germany), then transcardially perfused with 0.9 M NaCl solution, followed by 4% PFA in 0.1 M sodium phosphate buffer (PB) (pH 7.2). Brains were removed and stored in fixative for one to two days at 4°C. Coronal and sagittal brain vibratome sections (35–50 μm) were prepared. For background fluorescence reduction, slices were treated with 1% NaBH_4_ in PB for 15 minutes at RT with mild agitation. After four washing steps with PB, slices were incubated for one hour at RT with blocking solution: 10% FCS, 1% normal goat serum (NGS, Vector laboratoiers, Burlingame, CA, USA) 0.1% glycine, 0.1% lysine, and 0.1% Triton X‐100 in PB. Subsequent primary antibody incubation was carried out in the same solution overnight at 4°C with antibodies against LPA_1_ (1:200 to 1:500, Cayman Chemical, Ann Arbor, MI, USA) or LPA_2_ (1:100 to 1:250, generated by J. Aoki) and against GFAP, an astrocyte marker (1:500, Millipore, Billerica, MA, USA), or Tuj1, a neuroblast marker (1:500, Covance, Princeton, NJ, USA). After three washing steps with PB, slices were incubated with goat anti‐rabbit 488 or goat anti‐rat 488 and goat anti‐mouse 568 Alexa Fluor conjugated antibodies (1:1000; Molecular Probes, Eugene, OR, USA) in blocking solution (without Triton X‐100) overnight at 4°C for two hours at RT. Finally, brain slices were washed three times and coverslipped with Immu‐Mount (Thermo Fisher Scientific, Waltham, MA, USA) for further microscopic analysis.

### 5.9 Microscopy

Brightfield images of ISH and fluorescence images of immunohistochemical stainings of brain slices were captured with an IX83 inverted imaging system with a DP80 camera (Olympus, Shinjuku, Japan). Images were taken with either 20× UPlanSApo (0.75 NA) or 60× UPlanSApo (oil‐immersion, 1.35 NA) objectives. Confocal images of primary neurons and brain slices were acquired with upright laser microscopes (Leica DM 2500 and Leica SP8, Leica Microsystems, Wetzlar, Germany) equipped with a 63× objective (oil‐immersion, 1.2 NA) using sequential scanning with the 488‐nm line of an argon‐ion laser and the 543‐nm line from helium‐neon lasers (for Alexa 488 and Alexa 568, respectively). Background correction and adjustment of brightness and contrast were performed using either Leica confocal software (Leica Microsystems, Wetzlar, Germany), cellSens software (Olympus, Shinjuku, Japan), or ImageJ (NIH, Bethesda, MD, USA).

### 5.10 Quantitative real‐time PCR

C57BL/6 mice at different ages (E16–P30) were used for total RNA purification. The neocortex, hippocampus, cerebellum, and bulbus olfactorius were dissected and immediately snap‐frozen following harvesting from three sets of six animals of each age. The tissue was subsequently homogenized in TRIzol (Thermo Fisher Scientific, Waltham, MA, USA) reagent. Primary neurons, astrocytes, and microglia cells (approximately 3 × 10^5^ cells for each experiment from three independent preparations of three pregnant animals) were scraped in 1× PBS and centrifuged for five minutes at 900 × g at 4°C. Cell pellets were dissolved in 1 mL TRIzol reagent (Invitrogen, Eugene, OR), and the total RNA was purified according to the TRIzol protocol. RNA concentrations were determined using a UV/Vis spectrophotometer (Biomate 3 spectrophotometer, Thermo Fisher Scientific, Waltham, MA, USA). Subsequently, 2.5 μg total RNA was reverse‐transcribed to single‐stranded cDNA using the commercially available High‐Capacity cDNA Reverse Transcription Kit (Applied Biosystems, Foster City, CA, USA) according to the manufacturer's instructions. As a control, reaction was performed without MultiScribe reverse transcriptase. The quality of the amplified cDNA (with and without MultiScribe reverse transcriptase) was controlled by ß‐actin PCR.

The TaqMan Universal PCR Master Mix Kit was used for the TaqMan assay, and the reactions were performed in a 96‐well optical reaction plate from Applied Biosystems (Foster City, CA, USA). The following gene expression assays were employed: for LPA_1_ (assay ID: Mm00439145_m1), LPA_2_ (assay ID: Mm00469562_m1), LPA_3_ (assay ID: Mm01312593_m1), LPA_4_ (assay ID: Mm01228533_m1), LPA_5_ (assay ID: Mm01190818_m1), and LPA_6_ (assay ID: Mm00613050_s1 und Mm00519013_s1). GAPDH (glyceraldehyde‐3‐phosphate dehydrogenase, part no.: 4352932E), Pgk‐1 (phosphoglycerate kinase 1, assay ID: Mm00435617_m1), and HPRT (hypoxanthine guanine phosphoribosyl transferase, forward 5‐ATCATTATGCCGAGGATTTGGAA‐3; reverse 5‐TTGAGCACACAGAGGGCCA‐3; probe 5‐TGGACAGGACTGAAAGACTTGCTCGAGATG‐3, Metabion, Planegg, Germany) were used as internal controls to confirm the successful extraction of RNA, conversion to cDNA, and qRT‐PCR reaction. To determine the linear relationship between the threshold cycle (Ct) and the log starting copy number, serial dilutions of cDNA were amplified. In all experiments, the correlation coefficient was between 0.95 and 0.99. As a template for LPA_1_, LPA_2_, LPA_4_, and LPA_6_ assays, hippocampus cDNA was used; for LPA_3_ and LPA_5_ assays, spleen cDNA was used. To determine the relative gene expression in each experiment, samples were double‐tested and one “no template” control (NTC) was used. Quantitative RT‐PCR was mainly carried out using the ABI PRISM 7700 Sequence Detection System; only for analysis of oligodendrocytes, the ViiA 7 Real‐Time PCR System was used (both from Applied Biosystems, Foster City, CA, USA). The identity and purity of primary cells were shown by qRT‐ PCR with the neuroblast‐specific class III β‐tubulin (Tuj1) as a marker for neurons, the glial fibrillary acidic protein (GFAP) as a marker for astrocytes, the ionized calcium‐binding adaptor molecule 1 (Iba1) as a marker for microglial cells, the neuron‐glial antigen 2 (NG2) as a marker for immature oligodendrocytes, and the myelin basic protein (MBP) as a marker for mature oligodendrocytes (data not shown). All controls were included for all experimental time points (RNA quality, cDNA quantity and quality, qRT‐PCR quality) and each experimental sample, and were always negative.

### 5.11 In situ hybridization

ISH was performed on formalin‐fixed 8‐μm paraffin sections using the Advanced Cell Diagnostics RNAscope 2.5 HD Detection Kit (Bio‐Techne, Minneapolis, MI, USA). Briefly, C57Bl/6 mice were sacrificed by cervical dislocation, and brains were dissected and immersion‐fixed with 10% neutral buffered formaline for 16 to 32 hours at 4°C. Brains were dehydrated using a standard ethanol series, followed by xylene. Paraffin embedding was carried out overnight and 8‐μm sections were prepared with a sliding microtome. Sections were mounted on Superfrost Plus adhesion microscope slides (Thermo Fisher Scientific, Waltham, MA, USA) and dried overnight at RT. Brain sections were incubated with boiling target retrieval buffer for 15 minutes and then treated with protease plus for 30 minutes. Hybridization was performed following the manufacturer's protocol. The probes are as follows: Lpar1 (no. 318591), Lpar2 (no. 442691), Lpar4 (no. 318341), Lpar6 (no. 318351), and DapB (negative control). Nuclei were counterstained with hematoxylin.

### 5.12 Data Analysis

For quantitative comparison of the LPA receptor gene expression, data extracted from each qRT‐PCR run was analyzed using the 7500 Fast system software and the QuantStudio Real‐Time PCR software (both from Applied Biosystems, Foster City, CA, USA). The value of the noise fluorescence, usually indicated as the baseline of the run, was automatically determined. The Ct was automatically calculated and used to quantify the starting copy number of the target mRNA. Normalization of LPA receptor genes in brain tissue, astrocytes, microglia cells, and oligodendrocytes was evaluated to internal control of GAPDH and HPRT expression by means of the 2‐dCt method.^100^ Normalization of LPA gene expression in primary neurons was carried out in addition to internal control of Pgk‐1 using the same method.

### 5.13 Western blot analysis

Protein samples for western blots were obtained from whole brain of BalbC WT, LPA_1_‐KO, and LPA_2_‐KO mice. Tissue was homogenized in lysis buffer comprising 10 mM Tris‐HCl, pH 7.4, 10 mM EDTA, 1 mM phenylmethylsulfonyl fluoride, 1 μg/mL pepstatin A, 1 μg/mL leupeptin, 10 μg/mL soybean trypsin inhibitor, and 25 mM glycerophosphate as described.^101^ Afterward, the lysates were centrifuged for 10 minutes at 14.000 × g and 4°C. Protein extracts (40 μg) were boiled for 15 minutes at 60°C and separated on a 12% SDS‐gel. Blotting was carried out with nitrocellulose membrane BA 85 (Whatman, Maidstone, UK) using a Semi‐Dry Electro Blotter (PEQLAB, VWR International, Radnor, PA, USA) for 50 minutes at 14 V. Membranes were blocked for at least 1 hour at RT in 1× PBS buffer with 0.05% Tween 20 and 10% skim milk. The membranes were first incubated with anti‐LPA_1_ (1:500) or anti‐LPA_2_ (1:500) overnight at 4°C in the same buffer. Protein detection from LPA receptors overexpressing HEK293 cells lysate was carried out with anti‐HA, clone 3F10 (1:1000) (Roche Applied Science, Penzberg, Germany), or anti‐GFP (1:2000) (Abcam, Cambridge, UK). After washing three times with 1× Tris‐buffered saline (TBS) with 0.05% Tween20, membranes were incubated with secondary horseradish peroxidase conjugated antibody (1:5000, anti‐rabbit IgG or anti‐rat IgG) (GE Healthcare, Chicago, IL, USA) in 1× TBS, 0.05% Tween 20 overnight at 4°C. After washing, the immunoreaction was visualized using Pierce ECL Western Blotting Substrate on CL‐XPosure films. After exposure, the procedure of immunodetection was repeated, starting with blocking. Anti‐ß‐tubulin (1:2500) (Synaptic Systems, Göttingen, Germany), anti‐ß‐actin (1:20.000) (Sigma, St. Louis, MO, USA), or anti‐ATPase (1:500) (Abcam, Cambridge, UK) was incubated for 1 hour at RT in 1× TBS, 0.05% Tween 20; anti‐mouse IgG (1:5000) (GE Healthcare, Chicago, IL, USA) served as secondary antibody.

Other tested and not specific antibodies for immunodetection were rabbit polyclonal anti‐LPA_1_ (GeneTex Inc., Irvine, CA, USA), rabbit polyclonal anti‐LPA_1_ (LifeSpan Biosciences, Seattle, WA), rabbit polyclonal anti‐LPA_2_ (Novus Biologicals, Littleton, CO, USA), rabbit polyclonal anti‐LPA_2_ (Abgent, Heidelberg, Germany), rabbit polyclonal anti‐LPA_4_ (Acris Antibodies, Herford, Germany), and rabbit polyclonal anti‐LPA_4_ (Abcam, Cambridge, UK).

## CONFLICT OF INTEREST

The authors declare that they have no competing interests.
